# HIV prevalence and gender differences among new injection-drug-users in Tallinn, Estonia: A persisting problem in a stable high prevalence epidemic

**DOI:** 10.1371/journal.pone.0170956

**Published:** 2017-02-02

**Authors:** Anneli Uusküla, Mait Raag, Kristina Marsh, Ave Talu, Sigrid Vorobjov, Don Des Jarlais

**Affiliations:** 1 Department of Family Medicine and Public Health, University of Tartu, Tartu, Estonia; 2 National Institute for Health Development, Tallinn, Estonia; 3 The Baron Edmond de Rothschild Chemical Dependency Institute, Mount Sinai Beth Israel Medical Center, New York, New York, United States of America; Asociacion Civil Impacta Salud y Educacion, PERU

## Abstract

**Introduction:**

New injectors / younger drug users are an important population to target for intervention because they are often at especially high risk of HIV and HCV infection. We examined HIV prevalence and gender differences in HIV prevalence and risk behavior among new injection-drug-users in Tallinn, Estonia.

**Methods:**

Respondent driven sampling (RDS) interview surveys and HIV testing were conducted in Tallinn in 2009, 2011 and 2013. We classified “new injectors” as persons who reported their first injection as occurring within three years of the study interview. Recruiting trees of the three individual RDS studies were joined to form one RDS dataset and RDS estimates for prevalence and means were derived. Bootstrap tests were used to compare data from men and women, HIV infected and uninfected.

**Results:**

Among 110 new injectors (34 women and 76 men) the mean age was 24.5 (SD 7.5) years; 63% reported injecting mainly fentanyl, 34% injecting mainly amphetamine, 36% sharing syringes, 89% were sexually active, and, of these, 88% did not always use condoms in the last 6 months. HIV prevalence was 18% (95%CI 8–28%) (41% (95%CI 19–63%) among female and 7% (95%CI 2–12%) among male new injectors). Based on self-reports, 8.1% of all new injectors (and 22% of female new injectors) were HIV positive before starting to inject drugs. 40% of HIV infected reported receiving antiretroviral therapy. In multivariable analysis, gender (male: OR 0.12, 95% CI 0.03–0.45), main drug injected (fentanyl: OR 6.7, 95% CI 1.3–35.7) and syringe sharing (distributive: OR 0.11, 95% CI 0.02–0.55; and receptive: OR 3.7, 95% CI 1.0–13.5) were associated with the HIV seropositivity.

**Conclusions:**

New injectors exhibit high-risk behavior and correspondingly high HIV prevalence. Sexual transmission of HIV infection, including before injection initiation, is likely to be a significant contributor to HIV risk among female new injectors. This highlights the need to identify and target new injectors and their partners with gender specific interventions in addition to interventions to reduce initiation into injecting and ensuring provision of ART to HIV positive new injectors.

## Introduction

Young people aged 15–24 years accounted for an estimated 35% of all new HIV infections in 2012 [1]. Those who have recently begun injecting illicit drugs (“new injectors”) are typically young, engage in very high rates of injecting and sexual risk behavior [2–5], and are relatively unlikely to engage in evidence-based prevention initiatives such as needle syringe programs (NSP), medically assisted drug treatment (MAT) or antiretroviral therapy (ART) [6]. Because of these factors, new injectors are at especially high risk of acquiring hepatitis C (HCV) infection in almost all illicit drug-injecting populations [7,8] and are also at great risk of acquiring HIV in areas of high HIV prevalence. It has also been suggested that sexual behavior patterns may contribute to HIV transmission among new injectors [9–11].

A recent systematic review concluded that the HIV prevalence among women who inject drugs (WWID) in high-seroprevalence settings is higher than that for men (although the effect sizes varied considerably across different locations) [12]. As not all studies report higher HIV prevalence among female injectors [13], it is possible that higher HIV prevalence among women is more likely in long-duration high prevalence HIV epidemics. In addition, some studies have identified lower access to ART among WWID [14,15]. The potential reasons why WWID may be at higher risk of acquiring HIV than men who inject drugs are complex [16]. Importantly, WWID might be at higher risk of acquiring HIV through sexual transmission due to the more efficient male to female sexual transmission of HIV [17], and through having very high-risk sex partners (both for non-commercial and commercial sex partners). While there are data available on the gender differences on drug use and sexual behaviour among PWID [18,19], the data on use of prevention services (needle and syringe programs) by gender is rather limited.

As most studies of HIV among PWID have relatively modest numbers or new injectors and very modest numbers of female new injectors, there is comparatively little data on gender differences in HIV risk among new injectors. To expand on previous work involving HIV risk factors and gender specific characteristics among new injectors in high HIV prevalence settings, the current study examined new injection-drug-users in Tallinn, Estonia, and we report on HIV prevalence, and related risk and health behaviors among new injectors.

## Background

Estonia is a small country in the north-eastern part of Europe with a population of about 1,340,000 [20]. A very rapid HIV epidemic occurred in Estonia in 2000, and although HIV incidence has gradually decreased since the peak in early 2000s (from 108.0 / 100 000 in 2001 [21] to 20.6 in 2014 [22]), by 2014 Estonia still had the third highest per capita HIV incidence in Europe (22.1 / 100 000), after Ukraine (36.9 / 100 000) and Russia (58.4 / 100 000 in 2014) [22].

According to a global review of injection drug use and HIV epidemiology, Estonia has among of the highest prevalence of people who inject drugs (PWID) among persons in the 15–64 year age group (1.5% in 2007) coupled with a very high HIV prevalence among PWID [23]. Studies conducted among PWID in Estonia have shown a high prevalence of HIV of 40–90%. [24–27].

As described in detail previously [28], “Estonia’s capacity to manage its response to HIV and AIDS has increased greatly over the past decade”. Based on the numbers of syringes distributed and the estimated numbers of PWID in Tallinn, it was estimated that about 120 syringes per PWID have been distributed in Tallinn each year since 2008 [28]. Further, the proportion of PWID receiving ART in Tallinn has increased substantially over the years, reaching 50–60% among HIV-infected PWID in 2013 [28]. HIV prevalence has declined slightly and the (modelled) incidence has decreased rapidly—from 9.5/100 person-years in 2005 to 3.7/100 person-years in 2011 [29]. Data on HIV prevalence among PWID from a series of cross sectional studies in Tallinn (the HIV prevalence being approximately 50% since 2009) and decline in the numbers of new HIV cases detected among PWID at the HIV testing sites indicate stabilisation (or modest decline) of the HIV epidemic within this population group [29,30].

## Materials and methods

We defined “new injectors” as people who reported their first injection as occurring within three years of the study interview. Studies using duration of injecting typically define new injectors as people injecting of up to 3 years or less than 5 years [31]. The DUIT study, which is the largest interventional study to date on prevention of HCV infection, used a cut-off age of 30 for “young injectors" [32]. The overlap between chronological age (< 30 years) and short duration of injecting drugs (the first injection occurring within three years of the study interview) among our subjects was 90%, therefore our results may be compared with other studies with slightly differing definitions of new / young injectors.

Data for the current analysis were collected in a series of cross-sectional studies (n = 3, conducted biannually from 2009 to 2013) in Tallinn using standardized methods to subject recruitment and for behavioral and biological data collection. Detailed descriptions of the studies and methods used in analysis have been published elsewhere: Uusküla et al. 2011, 2010, 2015 [24,27,28]. These surveys used respondent driven sampling (RDS) for recruitment. Potential participants were eligible to be included in the study if they were at least 18 years of age, were Estonian or Russian language speakers, reported having injected in the previous two months, and were able and willing to provide informed consent. Recruitment began with the non-random selection of ‘seeds’ (n = 8, 2009; n = 6 in 2011 and 2013) purposefully selected (amongst PWID know to field team) to represent diverse PWID types (by age, gender, ethnicity, main type of drug used, and HIV status). After they had participated in the study, subjects were provided with coupons for recruiting up to three of their peers (other persons who inject drugs). Coupons were uniquely coded to link participants to their survey responses and to biological specimens, and for monitoring who recruited whom. Participants who completed the study received a primary incentive (a grocery store voucher with the value of 10 euros) for participation in the study and a secondary incentive (a grocery store voucher with the value of 5 euros) for each peer recruited (peers had come to study site, be eligible and complete study procedures for recruiter to receive the incentive).

We used an interviewer-administered questionnaire based on the WHO Drug Injecting Study Phase II survey [33] that elicited information on respondents’ demographics, injection drug use (including age at first injection), and sexual and related risk behaviors. Additional questions elicited information on participants’ use of various HIV-related services, including being tested for HIV (including the date (month/year) of the first positive HIV test result), self-report of HIV status, sources of new syringes, whether they were currently receiving methadone treatment, and for self-reported HIV-positive respondents, whether they were currently receiving ART.

Venous blood was collected from participants and tested for the presence of HIV antibodies using commercially available test kits (HIV-1/HIV-2 III Plus from Abbott Laboratories (Abbott Park, Illinois, USA) in 2009; and ADVIA Centaur CHIV Ag/Ab Combo (SIEMENS) in 2011, 2013).

Study participation in two studies (2009, 2011) was anonymous. The study protocol included pre- and post-HIV test counseling for study participants.

### Statistical analysis

To avoid multiple participation by the same “new injector” subjects in the different samples with anonymous participation (2009 and 2011) a combination of biometric measures of each respondent (circumference of each wrist and length of each forearm from elbow to middle finger +- 0.5 cm) and selected personal characteristics (gender, ethnicity, month of birth, year of birth, country of birth) was used. Using this method, we ascertained that no new injectors participated in both 2009 and 2011. In 2013, study participation was not anonymous and biometric measures were not collected. To avoid potential duplication of individual subjects in the 2011 and 2013 samples, a set of selected personal characteristics (gender, ethnicity, month of birth, year of birth, country of birth, age at first injection drug use) was checked and we identified 1 new injector who possibly participated in both 2011 and 2013. This subject was randomly allocated only to one of the years (2013) for the subsequent analysis. As persons classified as “new injectors” in the 2009 sample would not have been new injectors in 2013, eliminating potential duplicate participation in these samples was not needed.

Recruiting trees of the three individual RDS studies were joined to form one RDS dataset preserving the original recruitment chains. The structure of the joint RDS data was similar to that of an individual RDS study, the number of seeds equalling the sum of the number of seeds of the three studies. Based on personal characteristics there was only one person who probably had been recruited in 2011 and 2013; however, he had not recruited anyone in 2011 so omitting this subject from the 2011 dataset did not affect recruitment chains. The data collected in the process of recruiting the study subjects (i.e. the number of potential participants that the respondent knew within the target population and the coupon numbers of each respondent and his/her recruiter from the recruiting coupons) were used to derive RDS sequential sampling estimates for the mean value or the prevalence (with 95% CIs) for the variables of interest [34]. Bootstrap tests [35] were used to compare men and women.

Homophily is used to measure the degree to which respondents in a group recruited persons with characteristics similar to themselves rather than randomly from the entire population. [36]. For a specific characteristic, a homophily index of H = 1.0 (100% homophily) or index of H = −1.0 (100% heterophily) would indicate that all recruitment ties are formed with other members having the same value on the characteristic or with members having the opposite value on the characteristic, respectively.

Associations of HIV seropositivity were examined using multivariable logistic regression (using backwards elimination), from which adjusted ORs with corresponding 95% CIs, were estimated. Factors significantly associated with the outcome at an α level of 0.2 in a bivariable analysis were included in the multivariable model.

We used statistical environment R [37] with packages RDS [38] and RDS Analyst [39] for analyses. Sample proportions and RDS weighted estimates are presented in Table 1 (RDS estimates are presented in the Results section).

**Table 1:** The tendency of a group to recruit only others in the same group (homophily for key variables) among new injectors in Tallinn, Estonia (in 2009, 2011, 2013).

Variable	Homophily index
New injector	-0.020
Old injectors	0.292
Sex (male)	0.138
Sex (female)	0.028
Main drug injected (fentanyl)	0.326
Main drug injected (other)	0.274
HIV seropositive	0.231
HIV seronegative	-0.016
Sharing (last 6 months)	0.204
Not sharing (last 6 months)	-0.095

Ethical approval was obtained from the Ethics Review Board of the University of Tartu and the Beth Israel Medical Center Institutional Review Board for 2009–13 in New York, USA. Written informed consent was secured from all participants.

## Results

**Fig 1:**
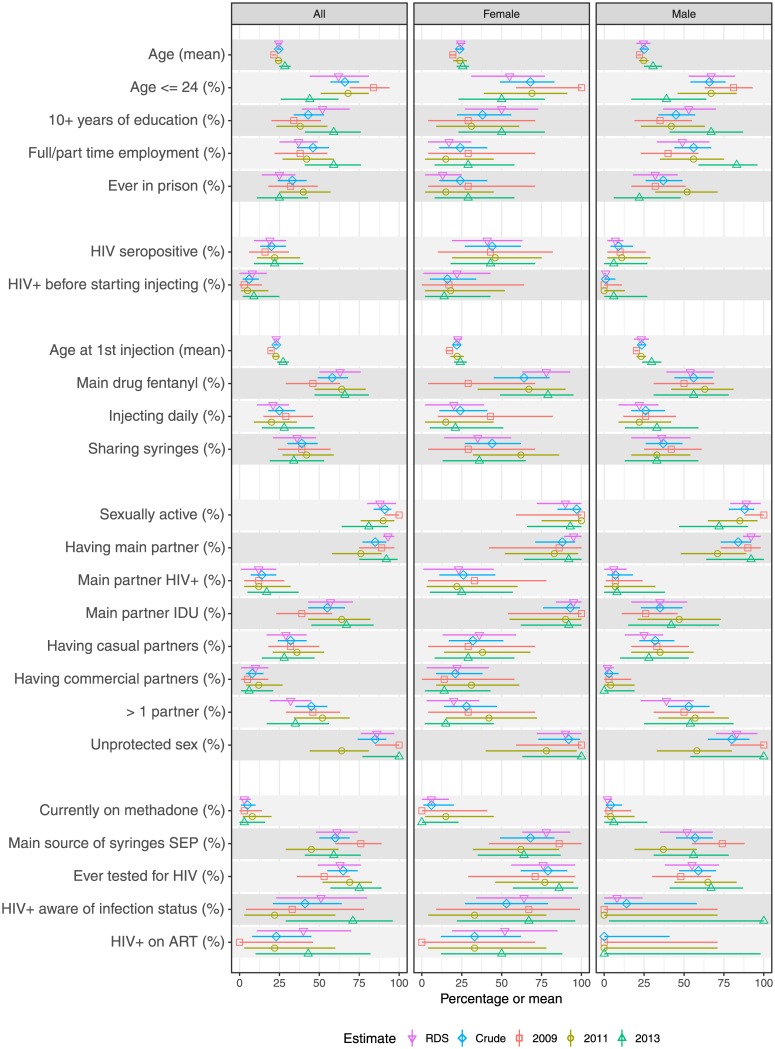
Socio-demographic, injection drug use, sexual behavior, HIV prevalence and service utilization characteristics of new injectors in Tallinn, Estonia (in 2009, 2011, 2013) (sample and RDS proportions with 95% confidence intervals).

From the total sample of current IDUs (n = 325 in 2009, n = 349 in 2011, and n = 328 in 2013), 14% (n = 109) were new injectors (34 women and 75 men). The proportion of new female injectors among all new injectors varied from 18% in 2009 to 44% in 2013 (sample proportions). Data on study sample characteristics of the study sample (by year and gender) are presented in Fig 1. Overall, the crude estimates (sample proportions) did not significantly differ from the RDS adjusted estimates, nor were there clear trends in estimates over the years 2009, 2011 and 2013 in the selected variables (Fig 1). Estimates for homophily indexes for key variables in the study sample were close to zero, suggesting a single underlying population for each sample (Table 1). Estimates for homophily indexes for both new and long term injectors were close to zero, suggesting a single underlying population for each group.

Demographic Characteristics (Table 2): The mean age of new injectors was 24.6 (SD 7.5; sample median 22) years, ranging from 18 to 53 years. There were no differences between the female and male new injectors in terms of education. Women were less likely than men to report current employment (17% vs. 49%, p<0.05) or ever being in prison (13% vs. 32%, p<0.05).

**Table 2:** Selected characteristics by gender of new injectors in Tallinn, Estonia (in 2009, 2011, 2013) (RDS proportions with 95% confidence intervals).

	All (N = 110)		Women (n = 34)		Men (n = 76)		p-value **
	n	%, 95% CI *	n	%, 95% CI *	n	%, 95% CI *	
*Socio-demographi characteristics*							
Age (years, mean)	110	24.5 (23.1–26.0)	34	24.4 (21.6–27.2)	76	24.6 (20.2–29.0)	0,8746
Age (< = 30 years)	96	90% (83–96%)	30	92% (83–100%)	66	89% (80–97%)	0,1106
Education (10+ years)	47	52% (39–69%)	13	50% (27–73%)	34	53% (37–70%)	0,8166
Employment (yes, full/part time)	50	37% (25–49%)	8	17% (4–31%)	42	49% (33–66%)	0,0039
Ever in prison (yes)	36	25% (14–35%)	8	13% (2–25%)	28	32% (18–46%)	0,0497
*Injection drug use*							
Age at 1st injection drug use (year, mean)	110	23.0 (21.6–24.5)	34	22.5 (19.9–25.2)	76	23.3 (18.7–27.9)	0,6144
Main drug injected (fentanyl) (last 4 weeks)	62	63% (50–76%)	21	78% (63–93%)	41	54% (39–69%)	0,0411
Injection frequency (daily +) (last 4 weeks)	28	21% (11–31%)	8	20% (2–39%)	20	22% (9–34%)	0,8862
Syringes sharing (yes, last 6 months)	43	36% (21–48%)	15	35% (14–56%)	28	36% (17–54%)	0,9627
Receptive sharing (yes, last 6 months)	32	19% (11–27%)	13	24% (7–40%)	19	16% (7–26%)	0,4780
Receptive sharing with main sex partner (among those with main partner)	12	10% (2–17%)	8	19% (3–35%)	4	5% (0–11%)	0,0587
Distributive sharing sharing (yes, last 6 months)	32	27% (14–41%)	8	20% (1–39%)	24	32% (13–50%)	0,3772
Distributive sharing with main sex partner (yes, among those with main partner)	7	5% (0–10%)	4	7% (0–17%)	3	4% (0–10%)	0,5759
*Sexual behaviour (last 6 months)*							
Sexually active (having main/casual sex partners)	98	88% (80–98%)	33	90% (72–100%)	65	89% (79–98%)	0,8804
Main partner (yes)	82	93% (90–97%)	28	95% (89–100%)	54	92% (87–98%)	0,5032
Main partner HIV +	11	12% (1–23%)	7	23% (1–45%)	4	6% (0–14%)	0,1007
Main partner PWID	45	57% (43–71%)	26	95% (84–100%)	19	35% (17–52%)	0,0000
Casual partner(s) (yes)	35	29% (17–42%)	11	36% (13–59%)	24	25% (13–37%)	0,4322
Commercial partner(s) (yes)	9	10% (1–18%)	7	22% (3–42%)	2	2% (0–6%)	0,0131
More than 1 partner (yes)	43	32% (19–45%)	9	20% (3–36%)	34	39% (23–56%)	0,1072
Unprotected sex (yes)	55	86% (76–97%)	22	90% (72–100%)	33	83% (70–96%)	0,5208
*HIV infection*							
HIV seropositive	22	19% (9–29%)	15	41% (19–63%)	7	7% (2–12%)	0,0002
HIV + aware of infection status (of those HIV+)	9	51% (23–80%)	8	64% (34–94%)	1	8% (0–24%)	0,0043
HIV + test before starting to inject ***	6	8% (0–17%)	5	22% (1–43%)	1	1% (0–1%)	0,0132
*Services utilization*							
Currently on methadone (yes)	5	3% (0–7%)	2	6% (0–17%)	3	2% (0–5%)	0,5302
Main source of new syringes NSP (last 6 months) (yes)	66	61% (48–74%)	23	78% (63–93%)	43	52% (35–68%)	0,0297
Ever tested for HIV (yes)	71	63% (49–76%)	27	76% (56–96%)	44	55% (38–72%)	0,1304
Currently on ART (yes) (of those HIV+)	5	40% (11–70%)	5	52% (19–85%)	0	0%	0,0000

* RDS estimates;

** Women vs men;

*** Based on self report and recall

Drug Use and Injecting Risk among New Injectors (Table 2): There were no differences in age at first injection or injection frequency between men and women. However, female injectors were more likely to report injecting fentanyl as the main drug (78% vs 54%, p<0.05). We collected a detailed history of sharing behaviour. One third of respondents reported sharing syringes in the last 6 months. There were no major gender differences in reported frequencies of sharing (women vs. men; distributive sharing—20% vs. 32%, p = 0.37; receptive sharing– 24% vs. 16%, p = 0.48). However, there were significant differences in the detailed patterns of sharing—female new injectors were more likely to report receptive sharing with their main sexual partner (19% vs. 3%), also a higher proportion of female new injectors reported a syringe exchange program as their main source of new syringes (78% vs. 52%, p<0.05). Across the sample, those HIV seropositive and aware of their infected status (n = 9) were as likely as those unaware of their status (n = 12) to report receptive syringes sharing (43% vs. 20%, p = 0.15) but less likely to report distributive sharing (4% vs. 17%, p<0.0001).

Sexual Behavior (Table 2): There were no significant differences between men and women in the proportion of respondents who were sexually active (89% of the new injectors reported sex with either a main, casual or commercial partner within the last 6 months), the proportion reporting more than 1 sexual partner (32% among the sexually active) or casual sex partners (29% among the sexually active). Among those who were sexually active, unprotected sex was common (86% did not always use condoms). Nearly all female new injectors (95%) reported having an injection-drug-user as a main sexual partner (vs. 35% of men, p<0.01), and nearly one quarter (23%) of women reported that their main sexual partner was HIV infected. Female new injectors were significantly more likely to report engaging in commercial sex than male new injectors (22% vs. 2%, p<0.02).

HIV infection prevalence and testing (Table 2): HIV seroprevalence among female new injectors was high (41%, 95%CI 19–63%) and significantly higher than the prevalence among men (7%, 95%CI 2–12%). Based on participant self-reports, <1% (n = 1) of male and 22% (n = 5) of female new injectors were HIV positive before starting to inject drugs (of those five women, only one reported ever engaging in sex work). Half (52%) of the HIV-infected female new injectors were receiving ART (vs. none of the male new injectors). Importantly there were significant gender differences in accurate knowledge of HIV positive status: while 64% of HIV infected women were aware of their status, only 8% of men had correct knowledge of their HIV infected status (p<0.001).

In a multivariable analysis HIV seropositivity was associated with gender (male: OR 0.12, 95% CI 0.03–0.45), main drug injected (fentanyl: OR 6.7, 95% CI 1.3–35.7) and sharing (distributive: OR 0.11, 95% CI 0.02–0.55; and receptive: OR 3.7, 95% CI 1.0–13.5).

## Discussion

This study documented a high HIV prevalence among new injectors in Tallinn (one fifth of people reporting injecting drugs for less than three years were HIV infected) and corresponding high self-reported rates of risk behaviors (35% reported sharing syringes/needles and 86% unprotected sex). Our findings of high HIV prevalence and high rates of risk behavior among new injectors are in agreement with reports from other sites ([40] Dar es Salaam; [41] Ukraine; [42,43] USA), as is HIV seropositivity being associated with injection equipment sharing and fentanyl as a main injection drug used [24,26].

There are relatively few studies providing gender-specific HIV prevalence (or incidence) estimates for new injectors. In a study conducted in 5 American cities, HIV prevalence among young injectors (aged < = 30 years) was 2.8% and did not differ between male and female respondents [4]. In a study from Dar es Salaam, Tanzania [40] HIV prevalence among young injectors (aged < = 25 years) was 31%, with substantial gender differences (55% among women vs. 12% among men). The authors concluded that the higher risk for women stemmed from the interplay of injection and sexual risks (multiple partners, commercial sex) [40].

Several findings of new important information from this study warrant discussion. Our results highlight the potential role of sexual transmission of HIV due to both high-risk sexual behaviors (unprotected sex, commercial sex) and high-risk sexual partners’ profile among female new injectors. We found that HIV prevalence was unusually high among female new injection-drug-users (41% vs. 7% among male new injectors). Furthermore, it is important to note that one fifth of female new injectors (half of the HIV seropositive new injectors) reported that they were HIV positive before starting to inject, and this is likely to be an underestimate. We do not have data on the time and route of HIV transmission, but many of these women report having HIV-positive drug-injecting sexual partners and infrequent use of condoms. Thus, we can hypothesize that they probably became infected through sexual transmission. The female new injectors who were not infected before beginning to inject would be at high risk for both sexual transmission and injection-related transmission from their drug-injecting sexual partners. Therefore, female new injectors may be at extremely high risk of HIV because of unsafe sexual behaviors with HIV-positive male injectors before and after they begin injecting, and from unsafe injecting behavior with their HIV-positive sexual partners once they begin injecting. Again, other recent studies among young female drug injectors have suggested that personal relationships might influence the perceptions that dictate their behavior [3,16]

Further, we observed significant gender differences in the use of prevention and harm reduction services. An earlier study conducted in Tallinn concluded that PWID who used pharmacies as their main source of needles (as opposed to NSP) were at a less "advanced" stage of their injection career and had lower HIV prevalence than NSP users [44]. In this study, male new injectors were less likely to report NSP as the main source of new/clean syringes than women. Whether this reflects more limited financial resources among women (less than one fifth of the female new injectors reported having part/full time employment) or higher awareness of the availability of prevention services warrants further research. Somewhat in line with our results, a study of young injection drug users by Montgomery et al (2002) [45] concluded that while female new injectors reported needle sharing more frequently than men, they also reported more protective behaviors such as needle exchange use and carrying clean syringes. Still, the current syringe exchanges in Tallinn may not be sufficiently effective in reducing sexual transmission of HIV from male injectors to non-injecting female sexual partners or in reducing injection-related transmission among primary sexual partners.

There were striking sex differences in awareness of HIV status and in the likelihood of receiving ART for those who were HIV positive. None of the HIV-infected male new injectors were on ART and only one in ten were aware of their infection status. One possible reason for this is that women may receive HIV testing and ART in the context of antenatal care or when undergoing abortions (42% of the female study participants had children (data not shown)). It has been repeatedly documented that female PWID face many different barriers to HIV service access including police harassment, judgmental health personnel and a fear of losing their children; and that they are more stigmatized than their male counterparts [46]. Strategies for HIV testing vary across Europe, but widespread, unacceptably high rates of late diagnosis among women suggests that current testing strategies are not adequately reaching the female population. The only group of women that is specifically mentioned in HIV testing guidelines is pregnant women, for whom opt-out testing is often recommended [47]. (HIV testing is a mandatory component of the antenatal care provided in Estonia [48]).

A finding that HIV infected new injectors were more likely to report receptive and less likely to report distributive sharing of syringes warrants mentioning. Our data indicated that those aware of their HIV infected status were less engaged in distributive sharing (than those HIV infected unaware of their status). We would hypothesize that they do not want to infect others. Higher likelihood for reporting receptive syringe sharing and less distributive syringe sharing among HIV infected PWID has been reported before (46).

While homophilies are certainly imperfect measures of mixing, it is interesting that the homophily for new injectors did not show any preference for new injectors recruiting other new injectors. Thou, it would appear that new injectors in Tallinn associate with long-term injectors (who have very high HIV prevalence), and thus if the new injectors do engage in injecting risk behavior (or sexual risk behavior among females), they would be at relatively high risk for doing so with an HIV positive individual.

Our study has some limitations that should be noted. The cross-sectional design imposes well-known limits for causal inference, and the modest sample size increases the likelihood of Type II error. However, we used rather robust measures of health status (HIV seropositivity) and risk behavior. Other potential sources of bias associated with the sensitive and illegal behaviors under investigation are socially desirable responses and recall bias. While these biases might influence female and male study participants unevenly they seem unlikely to have caused the clear patterns observed in this study. Strengths of the study are focus on gender, young injectors, and using biological data.

## Conclusions

New injectors / younger drug users are an important target for additional interventions because they are often at high risk of acquiring HIV and HCV. There is a need for focused gender-specific HIV strategies for women, men, and new/young injectors in addition to interventions to reduce initiation into injecting and ensuring provision of ART to new injectors who are HIV positive.
